# Drug-Coated Balloon Treatment for Urethral Strictures: Is This the Future? A Review of the Current Literature

**DOI:** 10.3390/jcm14082854

**Published:** 2025-04-21

**Authors:** Konstantinos Kapriniotis, Ioannis Loufopoulos, Aikaterini Apostolopoulou, Paul C. B. Anderson, Efstathios Papaefstathiou

**Affiliations:** 1 Department of Urology, Whipps Cross Hospital, Barts Health NHS, London E11 1NR, UK; konstantinos.kapriniotis@nhs.net; 2 Urology Department, Ipswich Hospital, Health Rd, Ipswich IP4 5PD, UK; loufogian@gmail.com; 3 Laboratory of Hygiene, Social & Preventive Medicine and Medical Statistics, School of Medicine, Aristotle University of Thessaloniki, 54124 Thessaloniki, Greece; katapost@yahoo.gr; 4 Urology Department, Russell’s Hall Hospital DGFT, Pensnett Rd, Dudley DY1 2HQ, UK; 5 Urology Department 424 Military Hospital, Ring Road Efkarpia, 56429 Thessaloniki, Greece

**Keywords:** drug-coated balloon treatment, urethral stricture, endoscopic treatment

## Abstract

Urethral strictures significantly impact patients’ quality of life, with endoscopic treatments such as direct vision internal urethrotomy (DVIU) and dilatation showing high recurrence rates. Drug-coated balloon (DCB) treatment, which delivers paclitaxel locally after dilation, is an innovative, minimally invasive approach aimed at reducing fibrosis and stricture recurrence. Paclitaxel’s antiproliferative and antifibrotic properties inhibit excessive collagen deposition, improving long-term outcomes. DCB treatment is now included in guidelines for managing recurrent bulbar strictures less than 3 cm in length. Recent studies, including the ROBUST trials, have demonstrated the efficacy of Optilume in improving the International Prostate Symptom Score (IPSS) and maximum urinary flow rate (Qmax). DCB has also shown a significant reduction in reintervention rates compared with endoscopic treatments in long-term studies, confirming its safety profile. However, the durability of DCB in complex or longer strictures remains uncertain, and its role as a salvage therapy post-urethroplasty requires further investigation. DCB represents a promising, cost-effective advancement in managing recurrent bulbar urethral strictures, particularly for patients unsuitable for urethroplasty. Future research should focus on refining patient selection criteria and exploring indications for other anatomical sites.

## 1. Introduction

Urethral stricture is defined as a narrowing of the lumen of the anterior urethra due to fibrosis of the epithelium and the surrounding corpus spongiosum. In contrast, posterior urethral narrowing is less common, arises from distinct etiologies and pathophysiological mechanisms, and is typically referred to as posterior (prostatic) urethral stenosis [1]. Anterior urethral strictures are relatively prevalent, with an estimated incidence of 0.6%, and their frequency increases with advancing age [2]. These strictures may arise from idiopathic, iatrogenic, traumatic, autoimmune/inflammatory (e.g., lichen sclerosis), or infectious causes. Historically, gonococcal urethritis was a leading cause of urethral stricture; however, with the advent of antibiotic therapy, it has been nearly eradicated in well-resourced countries, with idiopathic and iatrogenic etiologies now predominating. Specific anatomical sites, such as the urethral meatus and distal penile urethra, are associated with particular risk factors, including lichen sclerosis and previous hypospadias surgery. Notably, bulbar strictures tend to present at younger ages compared with those in other anatomical locations, suggesting a potential congenital component [3].

Urethral strictures are commonly associated with lower urinary tract symptoms related to voiding dysfunction, urinary retention, bladder stone formation, and, in severe cases, overflow incontinence and renal failure. These strictures substantially impair patients’ quality of life and impose a significant economic burden on healthcare systems [4]. Management strategies for urethral strictures include endoscopic interventions such as dilation and direct vision internal urethrotomy (DVIU) as well as reconstructive urethral surgery (urethroplasty). The choice of treatment and its success rate depend on various factors, including etiology, anatomical location, length, complexity of the stricture, prior interventions, and surgical expertise. Reported outcomes of endoscopic treatment in clinical case series vary due to differences in the stricture characteristics and definitions of success. Generally, primary, short bulbar strictures (<2 cm) may be treated endoscopically with success rates approaching or exceeding 60%. However, most studies report follow-up durations of only 1–2 years [5,6]. Success rates decline markedly following a second endoscopic intervention and approach zero after a third attempt. Additionally, factors such as longer strictures, non-bulbar locations, and severely impaired maximum urinary flow before dilation are associated with poorer outcomes [7,8]. For recurrent or complex anterior strictures, urethroplasty remains the gold standard treatment, with long-term success rates typically exceeding 90%, depending on case-specific factors. However, urethroplasty is an invasive procedure requiring anesthetic fitness, hospitalization, and advanced surgical expertise [9].

## 2. Rationale for Drug-Coated Balloon (DCB) Treatment

Various pharmacological agents have been evaluated for their potential to reduce stricture recurrence when administered intralesionally during DVIU. However, findings have been inconsistent. In a randomized controlled trial (RCT), Tabassi et al. examined the effect of intralesional triamcinolone during DVIU and found no significant difference in recurrence rates compared with placebo, though recurrence-free intervals were longer in the triamcinolone group at a mean follow-up of nine months [10]. Similarly, Mazdak et al. reported favorable short-term outcomes with intralesional mitomycin C compared with placebo in a small RCT of 40 participants with short strictures (<1.5 cm) at six months follow-up [11]. Subsequent trials also demonstrated the potential benefit of mitomycin C [12]. Conversely, studies investigating hyaluronic acid injections following DVIU yielded conflicting results, with the most recent RCT by Chung et al. reporting promising outcomes at a short follow-up of 24 weeks [13]. Despite these findings, larger studies with extended follow-ups are required to determine the long-term efficacy of these agents in preventing stricture recurrence.

Despite the suboptimal long-term outcomes of urethral dilation and DVIU, particularly for recurrent strictures, endoscopic management remains the most widely utilized treatment worldwide due to its minimally invasive nature, ease of execution, and limited requirement for specialized expertise [14]. The Optilume™ drug-coated balloon (DCB; Urotronic, Plymouth, MN, USA) was developed to enhance the outcomes of endoscopic interventions and to provide an alternative to urethroplasty for definitive treatment in selected patients. This device combines the mechanical dilation of the stricture with localized, precise delivery of paclitaxel through an inflatable drug-coated balloon [15].

Paclitaxel is an antimitotic and antiproliferative agent widely employed in chemotherapy for various malignancies, including breast, ovarian, and non-small-cell lung cancer [16]. It exerts an antifibrotic effect by inhibiting transforming growth factor-beta (TGF-β) during wound healing. Its antiproliferative properties have been leveraged in percutaneous angioplasty, where paclitaxel-coated balloons and stents have been utilized for over two decades to prevent post-angioplasty restenosis [17]. In the treatment of urethral strictures, paclitaxel is topically applied to the stricture site in a standardized dose facilitated by the circumferential micro-fissures created during balloon dilation. The drug’s lipophilic nature and prolonged half-life (3–7 days) enable it to remain within the dilated stricture during wound healing, thereby inhibiting collagen formation [18,19]. Animal models have demonstrated reduced collagen deposition in subjects treated with paclitaxel analogs in experimental urethral stricture models [20].

## 3. Material and Methods

In order to explore further the technique, safety, efficacy, durability, and post-treatment considerations of drug-coated balloon (DCB) therapy, we conducted a narrative review of the current literature using databases including PubMed, Scopus, and Google Scholar. To ensure a comprehensive overview, we also screened the reference lists of key articles and performed supplementary independent searches. Efforts were made to include the majority of relevant publications available up to January 2025. During the preparation of this manuscript, the authors used the GenAI tool for the purposes of editing and proofreading. The authors have reviewed and edited the output.

## 4. Surgical Technique

### 4.1. Key Considerations

Optilume DCB dilation may be performed under local anesthesia in an outpatient setting or under general anesthesia in the operating room. The procedure should be conducted under direct cystoscopic visualization with or without fluoroscopic guidance. To optimize stricture delineation and precise balloon placement, combined cystoscopic and fluoroscopic imaging is recommended. The Optilume DCB is available in two lengths (3 cm and 5 cm) and four diameters (18 Fr, 24 Fr, 30 Fr, and 36 Fr). The choice of device size depends on the stricture length and the caliber of adjacent healthy urethra. The balloon should extend approximately 5–10 mm beyond the stricture on each side. The selected balloon diameter should slightly exceed that of the adjacent healthy urethra.

The procedure begins with the patient positioned in lithotomy and the C-arm fluoroscope set at a 45-degree angle to facilitate urethral imaging. A retrograde urethrogram is then performed. Under cystoscopic guidance, a hydrophilic guidewire (0.035″ or 0.038″) is advanced into the bladder. The inflation device of the Optilume drug-coated balloon (DCB) is emptied of air and filled with 20 mL of a 50:50 contrast solution. Once the protective sheath is removed, care is taken to avoid any further contact with the balloon surface. The DCB is then advanced over the guidewire under direct vision to the site of the stricture. Correct positioning is confirmed fluoroscopically, ensuring that the radiopaque markers of the balloon extend beyond the proximal and distal ends of the stricture. The balloon is inflated and maintained in position for five minutes. Following deflation and removal of the balloon, a final fluoroscopic image is obtained. The procedure concludes with the insertion of a small-caliber (12 Fr or 14 Fr) urethral catheter, which is typically left in place for 3 to 5 days.

### 4.2. Technical Considerations for the Procedure

- Pre-dilation of the stricture using an uncoated balloon, urethral dilators, or DVIU is routinely performed by some surgeons and has been described in the ROBUST study publications. However, this step is not obligatory unless the strictured lumen’s diameter is insufficient to accommodate the DCB [18,21]. The decision to perform pre-dilation or DVIU remains at the discretion of the operating surgeon.
- The procedure can be executed solely under cystoscopic visualization with saline as the inflation fluid instead of diluted contrast medium. However, fluoroscopic guidance is recommended, particularly during the early phase of a surgeon’s learning curve, as it enhances procedural precision.
- The DCB may also be advanced through the 6 Fr working channel of a rigid cystoscope. However, to minimize excessive contact, advancing the balloon alongside the scope is preferable.
- The most commonly used balloon size for bulbar urethral strictures is 30 Fr, which is more effective compared with 24 Fr. Ideally, the balloon diameter should approximate the caliber of the distal healthy urethra to ensure optimal dilation and minimize the risk of trauma.
- In cases of dense fibrotic strictures, inflation duration may exceed the recommended five minutes. As the rated burst pressure varies among balloon sizes, careful review of specifications is advised before inflation.
- Following balloon deflation, no further cystoscopic examination or retrograde urethrogram should be performed to prevent drug washout from the luminal surface [15,18,21].
- Patients are advised to use barrier contraception for one month to prevent paclitaxel exposure to their partner. If their partner is of childbearing potential, contraception should be extended to six months.

## 5. Efficacy, Safety, and Durability

The clinical outcomes of DCB treatment for urethral strictures reported in prospective and retrospective noncomparative studies and one comparative clinical trial over the last years highlight the potentially significant clinical benefits, economic advantages, and favorable safety profile. By integrating findings from different studies, including the ROBUST trials, real-world assessments, and an economic analysis, DCB treatment demonstrates promising efficacy and durability as opposed to traditional endoscopic management techniques and consistent safety across various patient populations.

### 5.1. Efficacy

The efficacy of DCBs has been evaluated across different studies, each highlighting improvements in patient-reported outcomes and objective parameters. The vast majority of the literature investigates the use of Optilume DCB in anterior bulbar urethral strictures, while very limited data exist regarding its efficiency in strictures at different anatomical locations. In the largest retrospective series published to date, including 238 patients with primarily recurrent bulbar strictures, Ballesteros Ruiz et al. demonstrated a statistically significant improvement in the mean International Prostate Symptom Score (IPSS) from 24 to 6 points at a median follow-up of 8 months [22]. The ROBUST III randomized placebo-controlled trial reported that 61% of patients treated with DCB experienced an improvement in IPSS at 2 years, with a reduction from 22.0 to 10.1 points (*p* < 0.001). The length of the stricture and the number of previous dilatations or type of previous endoscopic treatment do not seem to affect the outcome in the DCB group at 2 years [23]. Similarly, the prospective single cohort ROBUST I study documented a mean IPSS reduction from 25.2 points to 7.2 at 5 years of follow-up among the 53 patients with recurrent, short (<2 cm) bulbar strictures. The same study demonstrated that 5-year efficacy was significantly associated with the DCB diameter, as 30 Fr DCB showed better outcomes compared with the 24 Fr ones [24].

In addition, a study by Castellucci et al. involving 23 patients noted a significant reduction in IPSS from a mean of 20 to 6 over a short-term follow-up of 3 months [25]. Similar improvement was reported by DeLong et al. in the prospective single cohort ROBUST II study, in which patients with recurrent bulbar strictures (<3 cm) demonstrated a mean improvement in IPSS from 18.4 to 6.0 at 1 year, marking a 67% reduction [26]. Also, a growing number of reports presented in urological meetings are consistent with similar improvement rates as assessed by symptom questionnaires [27,28]. Overall, the aforementioned outcomes were summarized in a recent pooled meta-analysis, which reported a mean reduction of 13 points (95% CI −15.8 to −10.3; *p* < 0.0001) [29].

Objective urinary function, measured by maximum urinary flow rate (Qmax), also demonstrated consistent improvement across different studies. In the Optilume arm of the ROBUST III RCT, the mean Qmax increased from 7.6 mL/s at baseline to 12.6 mL/s at two years (*p* = 0.003), while ROBUST I documented a five-year improvement from 5.0 mL/s to 19.9 mL/s [23,24]. Risks of recurrence, such as stricture length ≥ 2 cm or history of ≥5 prior dilations, seem not to affect the improvement in urinary flow [23]. Castellucci et al. reported an increase in Qmax from 8.15 mL/s to 18.11 mL/s over 3 months, whereas Noor et al. demonstrated Qmax improvements from 10.5 mL/s to 18.2 mL/s (*p* < 0.001) within 30 days [25,28]. Finally, the pooled meta-analysis by Estaphanous et al. confirmed the aforementioned findings, reporting an average Qmax increase of 10.11 mL/s (95% CI: −15.8 to −10.3; *p* < 0.0001) in patients treated with DCB [29].

Finally, the ROBUST clinical trials were unique in evaluating the DCB treatment of recurrent bulbar strictures based on strictly anatomical success, defined as the ability to pass a 16 Fr flexible cystoscope or a 14 Fr catheter through the treated stricture. In the ROBUST II study, anatomical success was achieved in 73% of patients at 6 months [26]. Building upon these findings, the ROBUST III study reported similar results with success rates of 76% and 70% at 6 and 12 months, respectively [23].

### 5.2. Durability

There are limited long-term data assessing the effect of Optilume DCB on urethral strictures. The single-cohort ROBUST I study has reported the longest follow-up of patients to date, with 29 of the initial 53 patients being available for follow-up assessment at 5 years. The authors reported a 58% rate of functional success as defined in the study methods (>50% improvement in IPSS) at the end of the study, which was consistent with sustained significant improvement in Qmax (15 mL/s) and post-void residual (82 mL) compared with baseline (*p* < 0.01). Also, 71% of the participants who completed the study remained free from re-intervention at 5 years, with the majority of reinterventions occurring within the first year of treatment [24]. The rest of the studies published so far have significantly shorter follow-up periods. In the ROBUST III RCT, the authors reported a 77.8% reintervention-free rate at 2 years of follow-up compared with 23.6% in the control group at 12 months (*p* < 0.001), with continuing improvement in IPSS, Qmax, and post-void residual compared with baseline and no significant deterioration compared with 1-year follow-up [23].

### 5.3. Safety

The safety profile of DCB treatment has been consistently favorable across all studies, accounting for the previously mentioned limitation of short follow-up. The ROBUST III trial reported mild and transient complications such as hematuria (13.9%), dysuria (6.3–8.7%), and urinary tract infections (6.3%) [23]. In ROBUST I, no severe complications like urethral perforation or long-term morbidity were reported, and complications such as hematuria resolved spontaneously [24]. The ROBUST II study reported no treatment-related serious complications, and the safety findings were further corroborated by the economic evaluation conducted in the NHS, which found the procedure to be cost-effective with minimal adverse events [30].

Data on sexual function after DCB treatment primarily derive from the ROBUST trials [24,26]. Improvements in erectile function and overall satisfaction, as measured by the International Index of Erectile Function (IIEF) questionnaire, were reported one year post-treatment with DCB. However, no changes in orgasmic function were observed, nor were there reports of ejaculatory dysfunction or new-onset erectile dysfunction. Semen analysis indicated stable sperm concentration and total spermatozoa counts. It is noteworthy that paclitaxel is detectable in semen for up to 6 months post-treatment, with peak concentrations observed at 1 month. As such, protected sexual intercourse is recommended within this period [24]. Similar findings were reported in subsequent studies, with no significant changes in IIEF-5 scores or ejaculatory function [25,26]. Furthermore, a study involving 16 patients with radiotherapy-induced posterior urethral stenosis treated with DCB reported no incidences of de novo urinary incontinence during a 3-month follow-up period [31].

### 5.4. Repeat DCB Interventions

The prevalence of recurrence following treatment with DCB has been reported to be 19.17% across studies with follow-up durations ranging from 3 months to 5 years [29]. However, data regarding management strategies after DCB treatment failure remain limited. According to the American Urological Association (AUA) guidelines, DCB is contraindicated in patients previously treated with DCB [32]. While a limited number of studies have identified 12 cases where repeat DCB treatment was performed, specific outcomes for this subgroup of patients were not reported [21,27,33].

In the 5-year results of the ROBUST I study, three of the five DCB failures managed with repeat DCB procedures were reported as anatomical successes (lumen > 14 Fr) at 6 months post-retreatment, with one patient lost to follow-up and one exiting the study early. There were no further details about the management of the rest of the DCB failures in this cohort [24]. In addition, in the ROBUST II study, two of the four DCB failures were re-treated with second DCB dilatations, and the remaining two had urethroplasty. However, no details on the outcomes of the repeat procedures were provided [26]. Similarly, no information on the further management of DCB failures was included in the ROBUST III reports [23]. Finally, Mahenthiran et al. reported that two of their DCB treatment failures were treated with repeat DCB, whereas the remaining two had simple dilatation and graft urethroplasty, respectively, with no further follow-up information available [33].

### 5.5. Benefits and Limitations of Endoluminal Therapy

Endoluminal therapies such as DCB dilation offer several advantages over open surgical procedures, including minimal invasiveness, reduced morbidity, shorter recovery periods, and quicker return to daily activities. Economic analyses like that conducted by Kelly et al. suggest that DCB therapy could yield significant cost savings for healthcare systems like the NHS in England [30]. However, these therapies are not without limitations. DCB efficacy diminishes in patients with extensive fibrosis or strictures longer than 3 cm, as highlighted in studies and current EAU and AUA guidelines [32,34]. While adverse events are generally mild, refractory cases may necessitate more invasive interventions such as urethroplasty. Additionally, concerns about paclitaxel toxicity persist, given the drug’s detectability in semen for up to six months, warranting adherence to contraceptive precautions [24].

## 6. Discussion

The findings from the ROBUST III study and the OPEN study have significantly influenced contemporary guidelines from both the American Urological Association (AUA) and the European Association of Urology (EAU), which now include recommendations for the use of drug-coated balloon dilation (DCBD) in the management of recurrent bulbar urethral strictures less than 3 cm in length [23,32,34,35]. Specifically, the EAU guidelines offer a weak recommendation for the application of DCBD in patients with a history of at least two previous endoscopic urethral treatments when urethroplasty is not a feasible option [34]. Similarly, the AUA guidelines provide a grade B recommendation for DCBD as an adjunct to palliative endoscopic treatments without specifying the required number of prior interventions [32]. Typically, DCBD is performed following direct vision internal urethrotomy (DVIU) or urethral dilation.

The rationale underpinning these recommendations is grounded in evidence demonstrating that recurrent instrumentation for bulbar urethral strictures is associated with failure rates as high as 80% [36]. Furthermore, repeated endoscopic treatments can exacerbate stricture complexity and length, thereby complicating subsequent urethroplasty procedures [37]. The OPEN study, which compared DVIU to open urethroplasty in patients with bulbar urethral strictures less than 2 cm, observed no significant differences in patient-reported voiding symptoms over a two-year follow-up period. However, urethroplasty yielded superior outcomes, with an odds ratio (OR) for improved Qmax of 2.6 at 1 year, and a 48% reduction in the risk of additional treatment within the same timeframe [35].

The ROBUST III trial evaluated 79 patients with urethral strictures measuring less than 3 cm in length and a diameter less than 12Fr, comparing the outcomes of DCBD with those of urethral dilation or DVIU alone. At 2 years, 61% (38/62) of patients treated with DCBD achieved at least a 30% improvement in the International Prostate Symptom Score (IPSS), while 77.8% of the DCBD group required no re-intervention at 24 months, compared with 23.6% in the control group at 12 months [23]. The study found no statistically significant differences in stricture etiology between the groups, though there were notable differences in dilation sizes, with the control group dilated up to 24 Fr and the DCBD group up to 30 Fr. Additional limitations included unblinded surgeons selecting their preferred methods of dilation or DVIU, disclosure of group allocation to participants after six months, and reliance on follow-up data only from patients who completed the study rather than the entire initial cohort. Specifically, Kaplan–Meier analysis was employed to assess freedom from repeat intervention, with participants right-censored throughout the study period. The comparison between groups excluded patients who discontinued or were lost to follow-up, which accounted for 11 out of 79 participants (13.9%) in the first year and 30 out of 79 (37.9%) by the second year. The reasons for discontinuation were documented separately, with 15 patients being documented as treatment failure [23,38].

Further evidence supporting the efficacy of DCB is provided by the five-year outcomes of the ROBUST I study. This trial included 53 patients with urethral strictures treated with DCB, reporting functional success in 58% (25/43) of participants and a 71.7% rate of no re-intervention over five years. However, the study lacked a control arm, and outcomes were similarly limited to patients completing follow-up or reaching an endpoint rather than the initial cohort. A Kaplan–Meier curve was generated to evaluate freedom from reintervention, with patients censored at the time of loss to follow-up or withdrawal from the study. Over the 5-year period, only 31 out of 53 patients (58.4%) completed follow-up: 8 (11.7%) were lost to follow-up, 13 (24.5%) were classified as treatment failures, 3 (5.6%) discontinued due to adverse events, and 2 (3.7%) withdrew their consent. Outcomes were also influenced by variability in the dilation method and size. Notably, only 25% of patients dilated up to 24 Fr achieved functional success at 5 years compared with 76% of those dilated up to 30 Fr. Similar trends were observed in post-void residual volumes and Qmax [24]. These findings underscore the heterogeneity in the criteria used to define procedural success across studies.

While some studies define urethroplasty success based on urethral caliber (e.g., >14 Fr), others emphasize patient-reported outcomes or the need for reoperation, highlighting the ongoing lack of consensus in the field. In a study by Anderson et al., five different definitions of anterior urethroplasty failure were compared with evaluated postoperative outcomes. These definitions included the need for further intervention, recurrence of stricture observed on flexible cystoscopy with a lumen diameter of less than 17 Fr, reduced urinary flow rate, recurrence of symptoms based on patient-reported questionnaires, and failure as defined by any of these criteria. The study demonstrated that five-year success rates varied significantly, ranging from 75% when defined by the absence of reintervention to 37% when based on reduced flow rates. This lack of standardized definitions complicates the comparison of outcomes across different patient populations, intervention types, and meta-analyses [39].

A recent meta-analysis encompassing 7 studies and 457 participants reported a mean reduction of 13 points in the International Prostate Symptom Score (IPSS) and a 10.1 mL/s increase in Qmax following DCB therapy. The recurrence rate was 19.2% with dysuria, hematuria, and urinary tract infections representing the most common complications. No major adverse events were noted, reinforcing the favorable safety profile of DCB therapy. However, the authors identified the limited follow-up period and the absence of randomized trials as significant limitations of the meta-analysis [29].

An economic analysis evaluating the integration of Optilume into routine clinical practice demonstrated the cost-effectiveness of DCB over a five-year horizon compared with endoscopic management within the National Health Service (NHS) in England. The model incorporated treatment pathways including endoscopic management, urethroplasty as a second-line option, and DCB, along with associated recurrence rates and costs. The analysis revealed a cost saving of GBP 243 per patient compared with urethroplasty and GBP 2502 per patient compared with endoscopic management over 5 years. However, the authors emphasized that these findings were based on non-UK data from clinical trials and extrapolated projections for five-year outcomes [30]. Although this study demonstrated that the Optilume DCB may represent a cost-saving alternative within the NHS in England, these findings may not be directly applicable to healthcare systems in other countries outside the UK.

### 6.1. Urethroplasty as the Definitive Treatment

When DVIU or DCBD fails, both the EAU and AUA advocate for urethroplasty as the definitive intervention [32,34]. The AUA advises against repeated endoscopic procedures after initial failures, citing limited success rates and the potential for stricture complexity. Urethroplasty, in contrast, boasts success rates of 80% to 90% even after unsuccessful endoscopic interventions [32]. The OPEN trial demonstrated that urethroplasty reduces the risk of re-intervention by 48% and improves maximum urinary flow rate (Qmax) by a factor of 2.6 within 2 years [35].

For longer bulbar urethral strictures (≥2 cm), primary urethroplasty is preferred due to poor outcomes associated with DVIU or dilation, particularly for strictures exceeding 4 cm [7]. In these cases, success rates decline to approximately 20% [7], whereas buccal mucosa graft urethroplasty achieves success rates exceeding 80% [40]. In patients unsuitable for urethroplasty due to comorbidities or personal preference, endoscopic options combined with antifibrotic agents such as mitomycin C or hyaluronic acid–carboxymethylcellulose may be considered, though long-term efficacy data remain limited [11].

Current guidelines recommend urethroplasty as the primary treatment modality for patients experiencing failure of endoluminal interventions for urethral strictures [32,34]. Nevertheless, there is insufficient evidence to evaluate how paclitaxel might influence the healing process or the outcomes of urethroplasty in patients who elect this surgical intervention. Reports on urethroplasty following prior DCB treatment are scarce, with only a small cohort of patients documented, and outcomes in this population remain undefined [27,33]. Consequently, further clinical and histopathological investigations are essential to elucidate the outcomes of urethroplasty post-DCB treatment as well as the feasibility of utilizing DCB as a salvage therapy following failed urethroplasty.

### 6.2. Expanding Applications and Patient Subgroups

Currently, DCBD is indicated only for recurrent urethral strictures measuring less than 3 cm. Nonetheless, there are several potential future applications for this technique, including treatment of strictures at other sites and those with varied etiologies. While the existing body of evidence remains limited, DCBD has been employed for the treatment of penile urethral strictures. Although the FDA has approved DCBD treatment for use in penile urethral strictures, the American Urological Association (AUA) guidelines highlight insufficient evidence to fully support its application in this context [32].

Several studies have reported real-world data encompassing small series of more than 19 patients with urethral strictures located in the penile urethra, 25 in the membranous urethra, 9 in the prostatic urethra, and 62 in the bladder neck [21,22,27,28,33]. Some studies have also noted differences in recurrence rates depending on the stricture’s location [22,27]. Furthermore, real-world data have included patients with longer strictures, with reported lengths exceeding 7 cm, as well as over 141 treatment-naïve patients [22,27,33].

Regarding the etiology of strictures, DCBD treatment has been utilized in more than 112 patients with prior urethroplasty, 89 with prior radiotherapy, and 13 with balanitis xerotica obliterans [21,22,27,28,31,33]. Subgroup analyses from individual studies, however, have not demonstrated significant differences in recurrence rates among these groups. Additionally, two noteworthy cases of DCBD use in female patients with urethral strictures following urethral diverticulum excision have been documented [21,41]. In the first case, the patient was followed for six months without recurrence or immediate complications, whereas the second case experienced recurrence, though the duration of follow-up was not specified [21,41]. It is evident that DCBD has been applied across a broad spectrum of stricture types, locations, and etiologies. Nevertheless, the small patient cohorts in individual studies, the lack of randomized controlled trials, and the underreporting of subgroup outcomes limit the available evidence. These limitations constrain the generalizability of this method and hinder the expansion of current guideline recommendations.

### 6.3. Future Directions and Research Gaps

Future investigations should explore combinations of DCB with adjunctive treatments like mitomycin C or novel drug-eluting technologies. Long-term studies focusing on challenging subgroups—such as individuals with radiation-induced strictures or complex anterior urethral involvement—are essential to refine treatment protocols. Emerging pharmacological strategies have also garnered interest. Antifibrotic agents like captopril, rapamycin derivatives, and ROCK inhibitors have demonstrated potential in preclinical models by mitigating fibroblast activity and collagen deposition [42]. Clinical trials assessing these agents’ safety, efficacy, and delivery mechanisms may expand the therapeutic arsenal for urethral stricture disease.

## 7. Conclusions

DCB interventions represent a promising, minimally invasive option for managing recurrent bulbar urethral strictures under 3 cm, particularly in patients unsuitable for urethroplasty. While repeat DCB dilation may offer temporary relief, urethroplasty remains the gold standard treatment for recurrent or complex strictures. Ongoing research into pharmacological adjuncts, long-term outcomes, and urethroplasty performance after DCB failure will help optimize patient care in this evolving field.
